# Pressure-Tuning Raman Spectra of Diiodine Thioamide Compounds: Models for Antithyroid Drug Activity

**DOI:** 10.1155/BCA/2006/68542

**Published:** 2006-10-04

**Authors:** Ghada J. Corban, Constantinos Antoniadis, Sotiris K. Hadjikakou, Nick Hadjiliadis, Jin-Fang Meng, Ian S. Butler

**Affiliations:** ^1^Inorganic and Analytical Chemistry Section, Department of Chemistry, University of Ioannina, Ioannina 45110, Greece; ^2^Department of Chemistry, McGill University, 801 Sherbrooke Street West, Montreal, QC, Canada H3A 2K6

## Abstract

The pressure-tuning Raman spectra of five solid, diiodine heterocyclic thioamide compounds (mbztS)I_2_ (mbztS = *N*-methyl-2-mercaptobenzothiazole) (**1**); [(mbztS)_2_I]^+^[I_7_]^−^ (**2**); (pySH)I_2_ (pySH = 2-mercaptopyridine) (**3**); [(pySH)(pyS]^+^[I_3_]^−^ (**4**); (thpm)(I_2_)_2_ or possibly [(thpm)I_2_]^+^[I_3_]^−^ (thpm = 2-mercapto-3,4,5,6-tertahydropyrimidine (**5**) have been measured for pressures up to ∼ 50 kbar using a diamond-anvil cell. Compounds **1**, **4**, and **5** undergo pressure-induced phase transitions at ∼ 35, ∼ 25, and ∼ 32 kbar, respectively. Following the phase transition in **1**, the pressure dependences of the vibrational modes, which were originally located at 84, 111, and 161 cm^−1^ and are associated with the S^⋯^I–I linkage, are 2.08, 1.78, and 0.57 cm^−1^/kbar, respectively. These pressure dependences are typical of low-energy vibrations. The pressure-tuning FT-Raman results for the pairs of compounds **1** , **2**, **3**, and **4** are remarkably similar to each other suggesting that the compounds are most probably perturbed diiodide compounds rather than ionic ones. The Raman data for **5** show that it is best formulated as (thpm)(I_2_)_2_ rather than [(thpm)_2_I]^+^[I_3_]^−^.

## INTRODUCTION

Iodine chemistry is proving to be of considerable interest lately, in part, because of the discovery of low-temperature, semi- and super-conducting polyiodides, which quickly led to the deliberate doping of conjugated polymers with elemental iodine, and ultimately resulted in the award of the 2000 Nobel Prize in Chemistry to Professor Heeger et al for their research on these materials [1]. A second important field in which iodine chemistry plays a pivotal role is in the activity of antithyroid drugs and there is now a major research effort focused on determining structure-activity relationships of thioamides with iodine [2, 3]. The antithyroid drugs that are most commonly used today are the thioamide derivatives, 6-*n*-propyl-thiouracil, *N*-methyl-imidazoline-2-thione (methimazole), and 3-methyl-2-thioxo-4-imidazoline-1-carboxylate (carbimazole). For the past few years, we have been exploring the iodine chemistry of thioamides in an effort to bring about a clearer understanding of the interactions involved in antithyroid drug treatment. As part of this research, we have reported the results of a fundamental study on the effect of high external pressures (up to ∼ 50 kbar) on the ambient-temperature FT-Raman spectra of four solid, diiodine-heterocyclic thioamide compounds [4]. In the case of one of these compounds, [(bztzdtH)I_2_} · I_2_ (bztzdtH = benzothiazole-2-thione), we discovered that I_2_ disproportionation occurs with increasing pressure and I_3_^−^ ions are produced. In addition, empirical correlations were established between the wavenumber of the I−I stretching vibration, the I−I bond length, and the applied external pressure. In this present paper, we have extended these pressure-tuning Raman spectroscopic studies to an examination of a second series of five, solid diiodine-heterocyclic thioamide compounds: (mbztS)I_2_ (mbztS = *N*-methyl-2-mercaptobenzothiazole) (**1**); [(mbztS)_2_I]^+^[I_7_]^−^ (**2**); (pySH)I_2_ (pySH = 2-mercaptopyridine) (**3**); [(pySH)(pyS]^+^[I_3_]^−^ (**4**); (thpm)(I_2_)_2_ or [(thpm)I_2_]^+^[I_3_]^−^ (thpm = 2-mercapto-3,4,5,6-tertahydropyrimidine) (**5**). These systems are also of potential importance as model compounds for the development of structure-activity relationships associated with the interaction of antithyroid drugs with iodine. They contain iodine atoms in several different structural arrangements and three of them have already been characterized by single-crystal X-ray diffraction, namely, compounds **1** [5, 6], **2** [5–7], and **4** [8].

## EXPERIMENTAL

The diiodine-heterocyclic thioamides **1**–**5** were prepared according to the literature procedures [2, 5–8]. Complete details of the pressure-tuning FT-Raman measurements, including the ruby R_1_ fluorescence-pressure calibration procedure, have been described elsewhere [4, 9]. A diamond-anvil cell (High Pressure Diamond Optics, Inc, Tucson, Ariz, USA) was used to generate the applied pressures. FT-Raman spectra were recorded on a Bruker IFS-88 FT-IR spectrometer equipped with an FRA-105 Raman module connected via two 1 m photo-optic cables to a Nikon Optiphot-II optical microscope using a Nikon 20X super-long-range objective. A near-IR (Nd^3+^: YAG) laser, emitting at 1064.1 nm with a power ∼ 25 mW, was used to excite the Raman spectra and typically a 2.6 cm^−1^ spectral resolution was employed while collecting 1000 scans. The band positions are considered to be accurate to at least ±1 cm^−1^.

## RESULTS AND DISCUSSION

In our previous pressure-tuning FT-Raman work on diiodine thioamide compounds [4], we pointed out that electron charge-transfer between the S atom of a thioamide and diiodine will result in stabilization of the lone pair of electrons on the S atom by overlapping of the S donor orbital with the σ*-orbital of diiodine. This situation will lead to a lengthening of the thione double bond in the thioamide and subsequent bond formation between the S atom and diiodine, together with a concomitant lengthening of the I−I bond. It was also shown that there are direct empirical correlations between the S−I, C−S, and I−I bond distances, namely, if the S−I distances are shorter than the C−S ones are, then the I−I distances will be longer than normal and there will be strong electron donation and vice versa. The application of high external pressures to the diiodine thioamide compounds would, therefore, be expected to produce some significant changes in bond distances and particularly on the positions of the low-frequency modes associated with the iodine atoms. There have been only a few high-pressure vibrational studies reported on diiodine systems and these have involved chiefly oxides [10, 11] and sulfides [12]. The interaction of polyiodides with polyvinyl alcohols has also been investigated under high pressure [13]. In addition, the effect of high pressures on the Raman spectrum of solid diiodine itself has been the subject of several investigations [14, 15]. In the present work, the FT-Raman spectra of compounds **1**–**5** were first recorded under ambient conditions and then pressure-tuning FT-Raman studies were undertaken on each of the species. An excellent review of the Raman spectra expected for different diiodine compounds has been published recently by Deplano et al [16].

**Figure 1:**
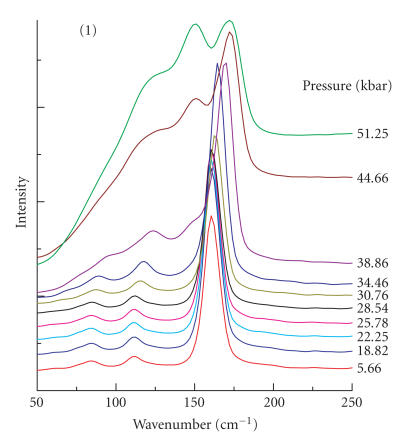
Pressure-tuning FT-Raman spectra of **1** in the low-energy region.

**Figure 2:**
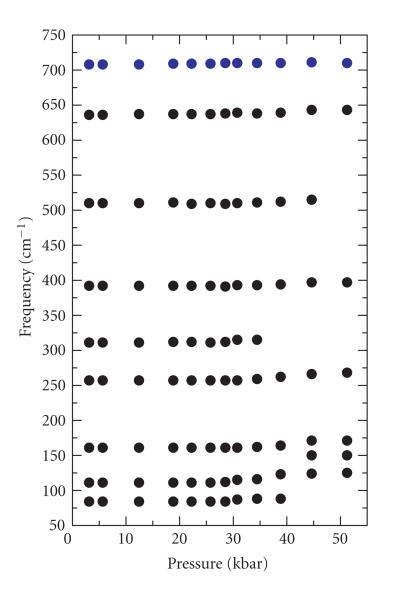
Wavenumber versus pressure plot for the FT-Raman spectra of **1** in the low-energy region.

FT-Raman spectra of compound **1** in the 50–250 cm^−1^ region are shown in Figure 1 for selected pressures up to ∼ 51.3 kbar. Initially, there are three bands observed at 84 w, 111 mw, and 161 vs cm^−1^. The Raman spectrum is closely similar to that reported for the (dtt)I_2_ (dtt = 1,3-ditholane-2-thione) for which the presence of a S^⋯^I−I linkage has been established [17]. Upon increasing the pressure, the three bands for compound **1** gradually shift to higher wavenumbers, as is typically the case [18]. At ∼ 35 kbar, the band originally at 84 cm^−1^ vanishes and a new band develops at ∼ 150 cm^−1^ which, eventually at ∼ 51 kbar, has a comparable intensity to that of the originally very strong peak, which has now shifted to 171 cm^−1^. These spectral changes can also be seen quite in the *ν* (cm^−1^) versus P (pressure, kbar) plot (Figure 2), which indicates that compound **1** undergoes a pressure-induced structural change at ∼ 35 kbar. From X-ray crystallographic data for 1 [5, 6], the I−I distance is 2.7912(9) Å which, from the linear plot of I−I bond length versus position of the *ν*(I−I) mode given in [4], would lead to an I−I stretching vibration being expected at ∼ 160 cm^−1^ at ambient pressure, just as it is observed for this adduct. Before the phase transition for compound **1** occurs at ∼ 35 kbar, the three vibrations originally located at 84, 111, and 161 cm^−1^ are completely pressure insensitive. Following the phase transition, however, the pressure dependences are 2.08, 1.78, and 0.57 cm^−1^/kbar, respectively, which are typical of low-energy vibrations. There are other weak Raman features detected at ambient pressure at 255, 311, 392, 511, 539, 636, and 708 cm^−1^, which are presumably associated chiefly with low-energy bending vibrations of the thioamide group. Furthermore, it is also worthwhile pointing out that the disappearance of the 311 cm^−1^ band of compound **1** at ∼ 35 kbar provides further evidence for the existence of the phase transition at this pressure.

In the case of compound **2**, it was initially thought that this would prove to be compound **1** with an additional I_2_ molecule attached to the S^⋯^I−I unit leading to a S^⋯^I−I−I−I linkage. However, X-ray crystallographic analysis has subsequently shown that this compound is actually [(mbztS)_2_I]^+^[I_7_]^−^ [5, 6]. The same compound was reported earlier by Demartin et al [7]. Three Raman bands are observed at ambient pressure at 85 w, 112 mw, and 161 vs cm^−1^. In addition, there are weak features at 257, 311, 391, 511, 539, and 637 cm^−1^. The Raman data are essentially identical to those of compound **1**. Upon application of pressure to **2**, the Raman spectra continue to be closely similar to those of **1**, including the existence of a pressure-induced structural change at ∼ 35 kbar. Therefore, from a vibrational standpoint, compounds **1** and **2** are essentially identical, not surprisingly since their formulations are really S · I_2_ and I^−^ · 3I_2_. Delplano et al [16] have emphasized the experimental difficulties inherent in measuring the laser Raman spectra of polyiodides, that is, some I_2_ may be lost during the measurements, for example, I_7_^−^ → I_5_^−^ + I_2_; → I_5_^−^ →→ I_3_^−^ + I_2_. There was no spectroscopic evidence, however, for the formation of any free I_2_ in this particular case—a strong Raman band would have been observed at ∼ 180 cm^−1^ [1, 14, 15].

**Figure 3:**
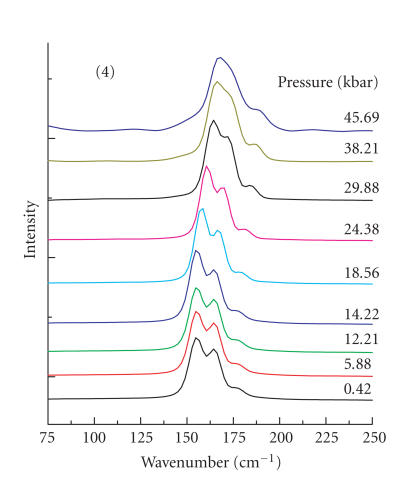
Pressure-tuning FT-Raman spectra of **4** in the low-energy region.

**Figure 4:**
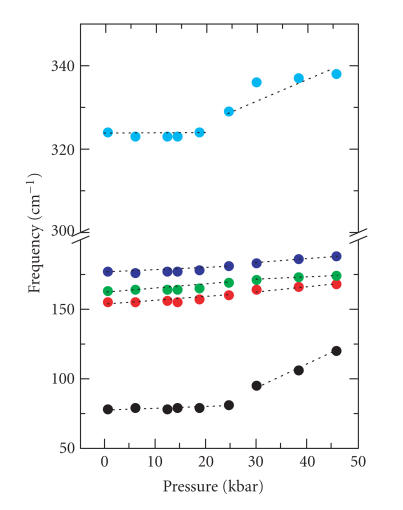
Wavenumber versus pressure plot for the FT-Raman spectra of 4 in the low-energy region.

The low-energy FT-Raman spectra of (pySH)I_2_ (pySH = 2-mercaptopyridine) (**3**) [(pySH)(pyS]^+^[I_3_]^−^ (**4**) are also quite similar to one another. It is possible that compound **3** may have disproportionated to **4** upon initial pressurization in the DAC, as has been reported previously for [(bztzdtH)I_2_} · I_2_ (bztzdtH = benzothiazole-2-thione) [4]. On the basis of X-ray crystallographic data for **4**, the I_3_ moiety is slightly bent. For a linear I_3_^−^ entity, only one very strong Raman band would be expected at ∼ 110 cm^−1^ from the symmetric inphase *ν*(I−I) stretching mode [17]. In the case of **4**, however, there is an intense doublet at 155 and 164 cm^−1^, and several other weaker features are observed at 77 sh, 110 vw, 176 sh, and 234 vvw cm^−1^. The appearance of this strong band at 164 cm^−1^, together with the observation of the other weaker bands, suggests that the I_3_ moiety in **4** is better formulated as an I^−^ · I_2_ entity, containing a slightly perturbed diiodine molecule, rather than as an I_3_^−^ species. A comparable situation exists for [(EtNH_2_)_2_dt]I_3_, which exhibits a very strong Raman band at 167 cm^−1^ and is considered to have a perturbed diiodine molecule [17]. The I^−^ · I_2_ formulation for **4** could also be another reason for the similarity of the spectra to those of **3**. The second intense Raman feature observed for **4** at 155 cm^−1^ may be the result of partial decomposition to another polyiodide species or possibly even site or factor-group splitting of the symmetric *ν*(I−I) vibrational mode. Under pressure, the doublet at 155 and 164 cm^−1^ shifts gradually to higher wavenumbers, with the higher energy component eventually merging with the lower energy one (Figure 3). The versus P plot in Figure 4 suggests that there is a pressure-induced structural change occurring at ∼ 25 kbar. The signals in the C−H stretching region are much too weak to be analytically useful at low pressures, but there is a significant increase in intensity occurring in this region, beginning just after the phase transition near 30 kbar, that continues until the high est pressure (∼ 46 kbar) is reached. This dramatic increase in intensity may be associated with pressure-induced fluorescence involving the I^−^ · I_2_ entity. Such pressure-induced fluores cence effects have been reported previously for diamond anvils [19], ZnS : Mn^2+^ nanoparticles [20], and acetone [21]. In addition, the acridine diiodine (2 : 3) adduct has been shown to a yellow-green fluorescence upon exposure to 632.8 He−Ne laser excitation [22].

**Figure 5:**
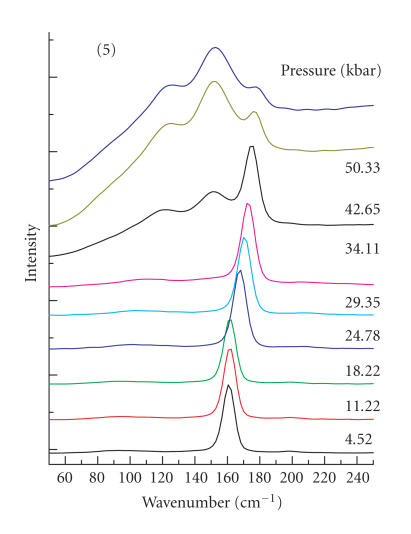
Pressure-tuning FT-Raman spectra of **5** in the low-energy region. The unlabeled pressure value is a repeated measurement of the experiment at 50.33 kbar and emphasizes the reproducibility of the pressure-tuning work.

**Figure 6:**
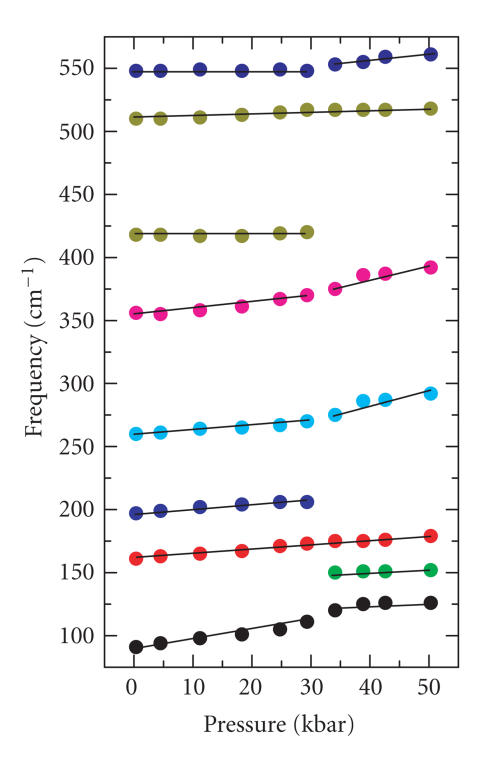
Wavenumber versus pressure plot for the FT-Raman spectra of **5** in the low-energy region.

Finally, a FT-Raman study was also conducted for compound **5**, which it was thought could be formulated as either (thpm)(I_2_)_2_ or [(thpm)I_2_]^+^[I_3_]^−^(thpm = 2-mercapto-3,4,5,6-tertahydropyrimidine). The principal, low-energy features appear at 91 w, 161 vvs and 197 w cm^−1^ suggesting the presence of neutral (thpm)(I_2_)_2_, containing perturbed diiodine molecules, rather than ionic [(thpm)I_2_]^+^[I_3_]^−^ for which the strongest Raman band would be expected at ∼ 110 cm^−1^ and not at ∼ 160 cm^−1^. Under pressure, this compound also undergoes a structural change at ∼ 32 kbar (Figures 5 and 6).

## CONCLUSIONS

Raman spectroscopy continues to be an extremely useful probe for examining the structural features of diiodine [14, 15, 23] and interhalogen [24] thioamide compounds. In the pressure-tuning FT-Raman spectroscopic work reported here, structural phase transitions were detected for compounds **1**, **4**, and **5** at ∼ 35, ∼ 25, and ∼ 32 kbar, respectively. There was no evidence of any free I_2_ formation for any of the compounds examined under high pressures. The similarity of the FT-Raman spectra of the two pairs of compounds, **1**, **2**, **3**, and **4**, may be the result of them being closely related perturbed diiodine complexes [25]. In the case of compounds **3** and **4**, it is also possible that a pressure-induced disproportionation of **3** may have occurred leading to the formation of **4**, in a similar manner to the effect of pressure on [(bztzdtH)I_2_} · I_2_ (bztzdtH = benzothiazole-2-thione) [4]. Finally, the FT-Raman data show that **5** is best formulated as (thpm)(I_2_)_2_ rather than the ionic species [(thpm)I_2_]^+^[I_3_]^−^.
